# Neuroimaging of depression with diffuse optical tomography during repetitive transcranial magnetic stimulation

**DOI:** 10.1038/s41598-021-86751-9

**Published:** 2021-04-01

**Authors:** Shixie Jiang, Jingyu Huang, Hao Yang, Ryan Wagoner, F. Andrew Kozel, Glenn Currier, Huabei Jiang

**Affiliations:** 1 Department of Psychiatry and Behavioral Neurosciences, University of South Florida, Tampa, FL USA; 2 Department of Medical Engineering, University of South Florida, 4202 E. Fowler Avenue, ENG 030, Tampa, FL USA; 3 Department of Behavioral Sciences and Social Medicine, Florida State University, Tallahassee, FL USA

**Keywords:** Depression, Psychiatric disorders

## Abstract

Repetitive transcranial magnetic stimulation (rTMS) is an effective and safe treatment for depression; however, its potential has likely been hindered due to non-optimized targeting, unclear ideal stimulation parameters, and lack of information regarding how the brain is physiologically responding during and after stimulation. While neuroimaging is ideal for obtaining such critical information, existing modalities have been limited due to poor resolutions, along with significant noise interference from the electromagnetic spectrum. In this study, we used a novel diffuse optical tomography (DOT) device in order to advance our understanding of the neurophysiological effects of rTMS in depression. Healthy and depressed subjects aged 18–70 were recruited. Treatment parameters were standardized with targeting of the left dorsolateral prefrontal cortex with a magnetic field intensity of 100% of motor threshold, pulse frequency of 10 per second, a 4 s stimulation time and a 26 s rest time. DOT imaging was simultaneously acquired from the contralateral dorsolateral prefrontal cortex. Six healthy and seven depressed subjects were included for final analysis. Hemoglobin changes and volumetric three-dimensional activation patterns were successfully captured. Depressed subjects were observed to have a delayed and less robust response to rTMS with a decreased volume of activation compared to healthy subjects. In this first-in-human study, we demonstrated the ability of DOT to safely and reliably capture and compare cortical response patterns to rTMS in depressed and healthy subjects. We introduced this emerging optical functional imaging modality as a novel approach to investigating targeting, new treatment parameters, and physiological effects of rTMS in depression.

## Introduction

Major depressive disorder is a chronic, recurrent, and debilitating mental health illness linked to significant functional impairment, disability, and mortality^1^. It is the primary cause of mental health-related disease burden globally, affecting approximately 300 million individuals^2^. Although there have been numerous clinical trials and studies on existing pharmacological and non-pharmacological options, its overall treatment is still challenging^3^. The largest and most extensive trial, the Sequenced Treatment Alternatives to Relieve Depression (STAR*D), revealed that antidepressants, even with augmentation, demonstrated suboptimal remission rates^4^. In lieu of medications then, a concerted effort has been dedicated to studying other treatment options, such as brain stimulation techniques. Transcranial magnetic stimulation (TMS) has now been established over the past decades as a safe and effective treatment for depression^5^.

Repetitive transcranial magnetic stimulation (TMS) was cleared by the Food and Drug Administration (FDA) for the treatment of depression in 2008^6^. When TMS is administered repeatedly at a specific frequency, it is referred to as rTMS. It involves passing an electrical current through an insulated coil, which creates an alternating magnetic field that penetrates the scalp and skull to induce neuronal depolarization and modulation^7^. Several landmark trials were conducted that reliably demonstrated its efficacy and safety in improving depressive symptoms^8–11^. However, despite its increasing use, the average response and remission rates have been modest at best. A previous meta-analysis of 29 studies and 1371 patients reported only a 29% average response rate and 19% average remission rate in randomized trials^12^. The effectiveness of TMS is likely reduced due to: (1) non-optimized targeting, (2) unclear ideal stimulation parameters (e.g., patterns, frequencies, dosage), and (3) a lack of understanding of how the brain is physiologically responding, during and after, stimulation^13^. As such, neuroimaging with rTMS has been a particularly promising area of research that has been pursued in order to answer these questions.

Brain imaging is theoretically able to offer valuable information regarding the above unanswered questions about improving TMS. However, due to the immediate brain changes that occur, only a few modalities possess the temporal resolution required to appropriately evaluate such conditions. Prior studies on brain activation and connectivity during TMS include the usage of functional magnetic resonance imaging (fMRI), magnetoencephalography (MEG), electroencephalography (EEG), and functional near-infrared spectroscopy (fNIRS)^14–17^. As fMRI, MEG, and EEG use signals that involve the electromagnetic spectrum, their overall resolution and quality are subject to significant measurement artifacts given the fact that TMS produces very strong magnetic and electrical fields. Due to this, fNIRS has been investigated for concurrent imaging as it measures an optical signal, which has no electromagnetic interference. It also has benefits of being portable, safe, cost-effective, and less restrictive than other devices. However, fNIRS has several technical limitations including scalp interference, shallow imaging depth, low spatial resolution, and an inability to produce three-dimensional images^18^. Our proposed solution to these problems involves a more novel neuroimaging technique called diffuse optical tomography.

Diffuse optical tomography (DOT) is an emerging noninvasive imaging modality based on the scattering and absorption properties of non-ionizing near-infrared light in biological tissue. It can be viewed as an extension and improvement to fNIRS, similar to the distinction between magnetic resonance spectroscopy and magnetic resonance imaging. Using multiple near-infrared wavelengths, it is able to accurately measure absolute and relative deoxygenated ([Hb]), oxygenated ([HbO]), and total hemoglobin ([HbT]) concentrations. Most pertinently, it combines multi-channel data acquisition with sophisticated image reconstruction algorithms to produce quantitative three-dimensional images of changes in regional blood volume and oxygenation at high temporal and spatial resolutions^19^. It overcomes many of the limitations of fNIRS due to taking advantage of multiple overlapping channels and a wider range of source-detector distances, along with inherently different reconstruction algorithms. This allows DOT to distinguish between hemodynamic changes at different and further depths and reduce interference from the scalp or skull^20^. Over the past decades, it has been successfully used clinically for imaging of epilepsy, breast cancer, osteoarthritis, and cortical activations^21–24^. Impressively, it has been demonstrated that DOT is able to detect hemodynamic responses equivalent to fMRI in terms of spatial resolution (within a depth of 5 cm)^25^. Similar to fNIRS, it also possesses the benefits of being portable, safe, and cost-effective.

The primary objective of this study was to introduce DOT as a safe, effective, and feasible option for conducting noninvasive, continuous functional imaging of the brain during and after rTMS in healthy and depressed individuals. We hypothesized that rTMS would produce cortical activation patterns that would be reliably captured by DOT. In addition, we theorized that the hemodynamic changes, especially in terms of volume, between healthy and depressed individuals may offer new insights into how neuroimaging can be further used to improve TMS parameters and our understanding of the physiologic effects of brain stimulation.

## Methods and materials

### Study sample

Eligible subjects included individuals with no previous or current history of a psychiatric disorder (healthy control group) and those with a current Diagnostic and Statistical Manual of Mental Disorders, 5th Edition (DSM-5) diagnosis of major depressive disorder (depressed group) aged 18–70. Exclusionary criteria for study participation included a previous history of psychosis, bipolar disorder, posttraumatic stress disorder, eating disorder, or obsessive–compulsive disorder; pregnancy; personal or immediate family history of a seizure disorder; presence of ferromagnetic material in the head, neurologic disorder, or medication capable of altering the seizure threshold; vagus nerve stimulation implant; or history of electroconvulsive therapy failure.

**Figure 1:**
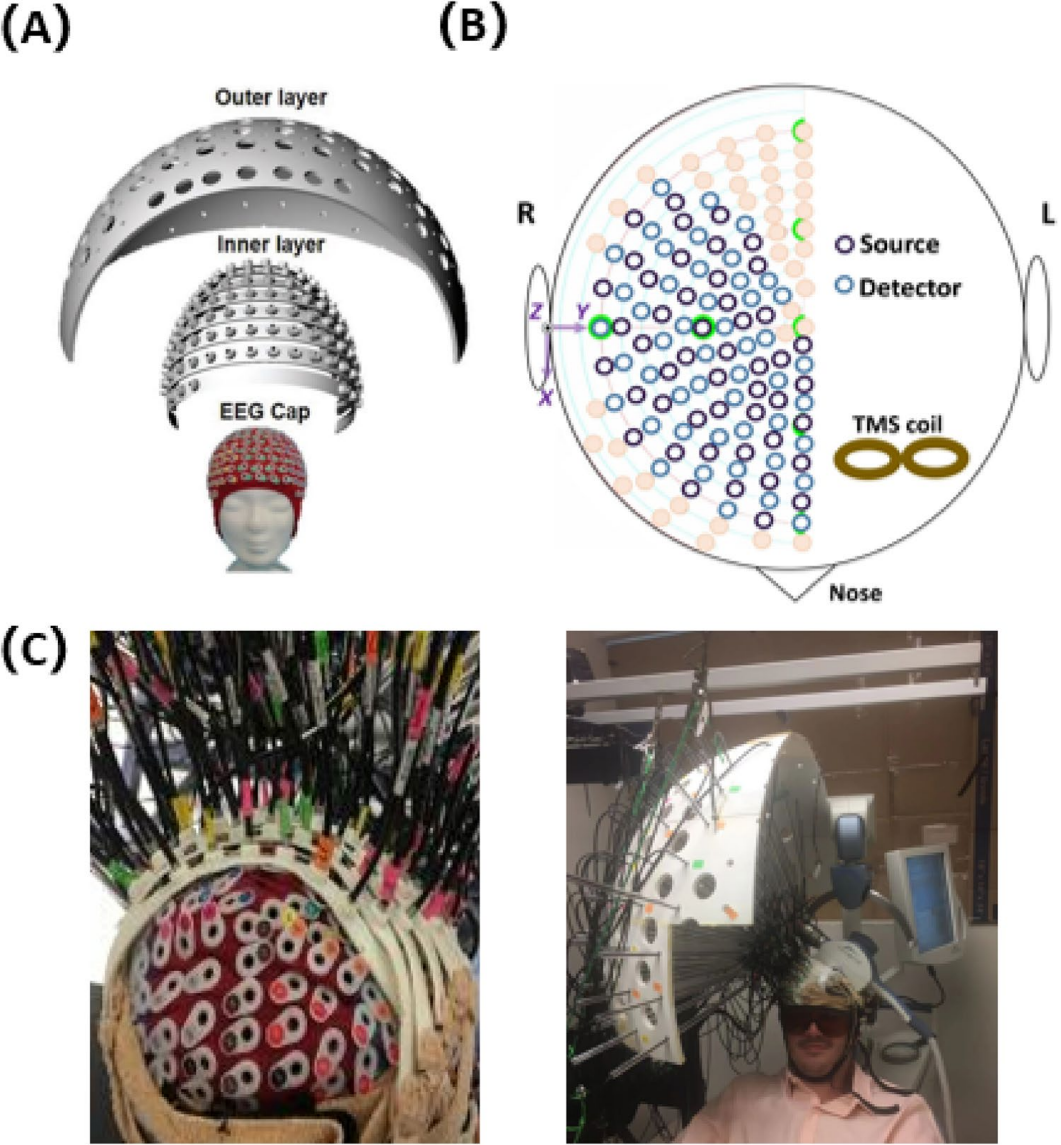
Custom DOT head interface. (**A**) Two layer design with an inner and outer layer superimposed upon a modified electroencephalogram cap; (**B**) top-down schematic depicting the location of the TMS coil (light brown figure of eight symbol) over the left dorsolateral prefrontal cortex and the DOT cap over the right hemisphere (centered upon the R DLPFC); (**C**) photographs of the interface connected to a human subject; the probe comprised of 48 source-detector pairs in total.

The research protocol was approved by the University of South Florida Institutional Review Board. All methods, including those involving humans, were conducted in accordance to standard protocols mandated by the Institutional Review Board. Informed written consent was appropriately obtained by each subject (depressed and healthy) prior to enrollment in the study. Additional informed written consent was also completed by one subject for permission to publish their image in any hard-copy, online, and/or open-access journal article (Fig. 1b). A physician screened participants with a Structured Clinical Interview for DSM-5^26^, Transcranial Magnetic Stimulation Adult Safety Screening Questionnaire^27^, a medical history review, and a physical exam. Routine laboratory studies including a complete blood count, complete metabolic panel, thyroid stimulating hormone, urine toxicology screen, urine pregnancy test (if the participant was a woman of child-bearing potential), and electrocardiogram were obtained during the screening process. Subjects were required to be medically stable before enrollment. All imaging and treatments were conducted in the same suite at the University of South Florida Neurotherapies Clinic.

### rTMS procedure

All rTMS treatments were performed with a Neurostar TMS Therapy System (Neuronetics, Inc., Malvern, PA, USA). Stimulation protocol was followed per the product documentation. Subjects were placed in a recliner and ear plugs were inserted to minimize possible hearing impairment from the TMS machine noise. The location of the motor strip was estimated by stimulating the cortex at low frequency (1 Hz) and device output (45%), advancing the power and repositioning the coil to elicit a reliable (5 out of 10 trials) muscle twitch of the abductor pollicis brevis in the appropriate contralateral hand^28^. Using the TMS Motor Threshold Assessment Tool, the motor threshold (MT) was determined four times and averaged^29^. Treatment parameters were standardized for each individual at the left dorsolateral prefrontal cortex (DLPFC) (Neurostar standards; 5.5 cm forward parasagitally from the identified point used to determine the MT) with a magnetic field intensity of 100% of MT, at a pulse frequency of 10 pulses per second, with a 4 s stimulation time and a 26 s inter-stimulation interval time for a total exposure of 3000 pulses per session.

### DOT procedure

All imaging was captured by a custom built fast multispectral DOT imaging system constructed from our laboratory’s previous studies^30^. The system involves a main computer sending a signal to a light emitting diode (LED) controller which activates two groups of LEDs (48 optodes per group; one group at 780 nm and the other at 850 nm) (High-power NIR LEDs, Epitex, Inc.). Two core boards (CuteDigi Technologies), consisting of an Altera EP2C8 FPGA, 50-megahertz crystal oscillator, and 139 input/output pins, were used to control the time sequences of each LED. The light beams were sequentially delivered to the measuring interface through fiber optical bundles. The subsequent diffusing light from the tissue was received by 48 highly sensitive avalanche photodiode detectors (APD C5460-01, 12-bit resolution, maximum analog–digital conversion rate of 1.25 mega-samples/s) and converted to electrical signals for further processing. Continuous-wave measurements of three-dimensional data were captured through the 48 pairs of highly sensitive photo source-detectors. A full set of high density tomographic optical data was acquired with a unit consisting of two PCI-DAS6071 boards at a 14.4 Hz collection rate for both the wavelengths.

Participants were positioned in the TMS device chair and adjusted to ensure comfort throughout the experimental procedure. We utilized a two-layer interface coupled with a 256-channel medium-sized EEG cap capable of fitting the head size of all of our subjects (Fig. 1). All external light was blocked from entering the clinical lab, and the ambient lighting was tested to ensure that it did not contaminate the DOT signal. The DOT probe, consisting of the 48 pairs of source-detectors, was then positioned over the right hemisphere (centered on the R DLPFC). The probe was fastened using velcro straps and attached against the scalp with the tips directly touching the skin for maximum efficiency of light transmission. Any hair strands under the probes were manually moved away using combs to facilitate optimal contact and avoid contamination by light absorption. The subjects were then instructed to sit quietly, remain awake, not move, and keep their eyes open. DOT data acquisition occurred 1 min prior to stimulation, during the 24 stimulation/rest epochs (30 s per epoch), and during a 2-min post stimulation period, resulting in a total recording time of 15 min. Images were only captured for a single treatment session.

### Image analysis and processing

The DOT data were processed and analyzed channel-wise (one channel represents one source-detector pair). The setup comprised of 48 channels in total. The raw data for each channel were inspected during the entire experiment time course in order to exclude epochs with significant discontinuity. Several channels had epochs with inadequate measurements due to suspected motion artifact, thus were removed accordingly. After ensuring the quality of the data, we used a band pass filter (cut off frequencies $fH$ = 9 Hz, $fL$ = 0.02 Hz) to exclude instrumental noise and saturated signals. Contamination of signal from peripheral co-stimulation during TMS is inherently prevented by the DOT reconstruction algorithms which include boundary limits when calculating the data from the high number of source-detector pairs. Skin pigmentation is also accounted for based on inherent DOT algorithms and adjustment of signal output and interface settings to prevent polluted data acquisition. Data were averaged for all participants and the resting time in the experiment was used as the baseline. The high-density tomographic data acquired at each wavelength was used to reconstruct a three-dimensional image of tissue absorption coefficient using a finite element-based algorithm previously developed and optimized^31^. The physiological measurements in terms of oxygenated and deoxygenated hemoglobin, [HbO] and [Hb], respectively, were calculated using the absorption coefficient images at both wavelengths and a modified Beer–Lambert law coupled with a least-square fitting procedure through pseudo-inverse matrix calculations^32^. Cerebral blood volume was estimated by calculating the total hemoglobin concentration, [HbT], which was achieved by summing [HbO] and [Hb].

### Definition of regions of interest

Regions of interest were identified on a MRIcron ch2.nii.gz MRI template. The coordinate system used was based on a Cartesian three-dimensional configuration combined with a 256 channel EEG system. The origin [0, 0, 0] was set at C18 on the EEG system. From this location, three-dimensional images including coronal, sagittal, and transverse views were derived. The direction of the X, Y, and Z axes were defined as right to left, posterior to anterior, and inferior to superior. This coordinate configuration was combined with an EEG system for ease of use and recognition purposes as EEG mapping is commonly employed in other protocols. The right prefrontal cortex was centered at coordinates [51, 35, 53] for the healthy subjects. For the depressed group, this location was centered at coordinates [48, 39, 57]. These coordinates were based on the averaged areas of maximum peak activations during image capturing and were within the expected range (Figure S1).

### DOT statistical analysis

Data analysis was conducted using MATLAB (version R 2018a) and SAS (9.4). Subject means were allowed to vary around an individualized intercept across trails with stimulation type as a within subject factor. Redundant analysis was conducted using repeated measures analysis of variance (ANOVA) to allow simpler interpretation of the results. The alpha level was set at 0.05 for all calculations.

## Results

### Participants

Eight healthy individuals (7 men and 1 woman) with a mean age of 33.5 ± 15.9 years and 11 depressed patients (2 men and 9 women) with a mean age of 51.8 ± 15.7 years were initially recruited as paid volunteers. Two healthy subjects were excluded due to significant motion artifacts that could not be rectified. One depressed subject was excluded due to weak signal processing and three others volitionally left the experiment prior to any stimulation or data collection due to personal life conflicts. In total, six healthy individuals (5 men and 1 woman) with a mean age of 36.3 ± 16.7 years and seven depressed individuals (2 men and 5 women) with a mean age of 49.1 ± 17.8 years were included for final analysis. All the participants were right-handed, as determined by the modified Edinburgh Handedness Questionnaire^33^, and had normal or corrected-to-normal vision. All subjects were of Caucasian descent except for two Asian females (one from each study group). For the depressed individuals, all of them had failed at least 3 antidepressants, been diagnosed by a psychiatrist for at least 3 years with Major Depressive Disorder, and remained on antidepressants while receiving rTMS treatment.

### DOT imaging data

**Figure 2:**
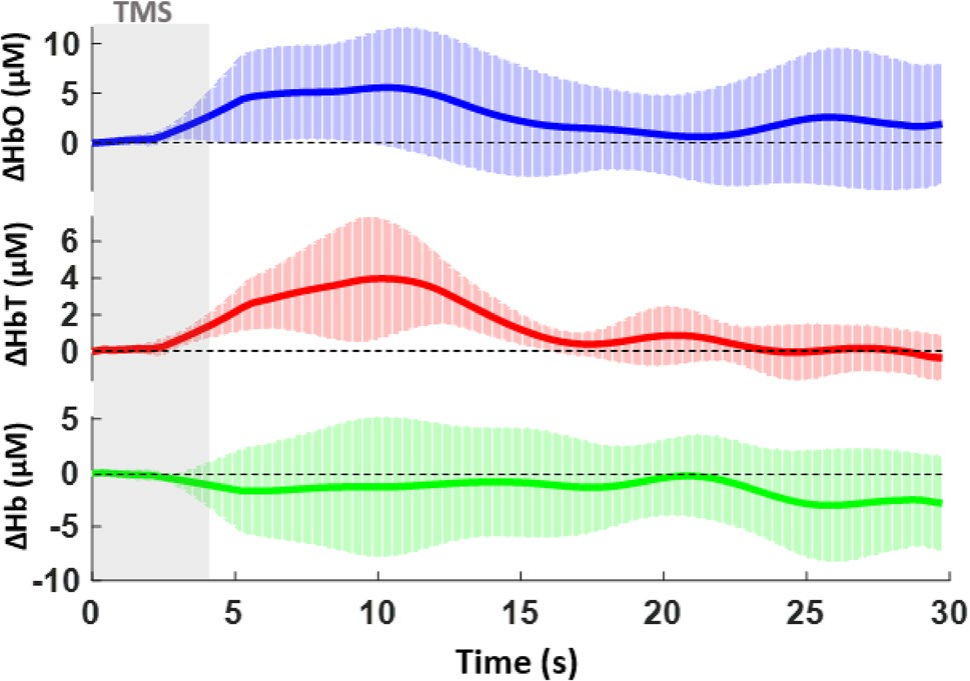
Averaged time course of oxygenated [HbO], total [HbT], and deoxygenated [Hb] hemoglobin signals in the healthy subjects for the contralateral or right dorsolateral prefrontal cortex. Ten hertz stimulation was performed for 4 s followed by 26 s of rest for a total of 12 epochs. The x-axis represents the time from 0 to 30 s during the epoch and the y-axis represents the mean and standard deviation for relative hemoglobin concentration in µM/L.

**Figure 3:**
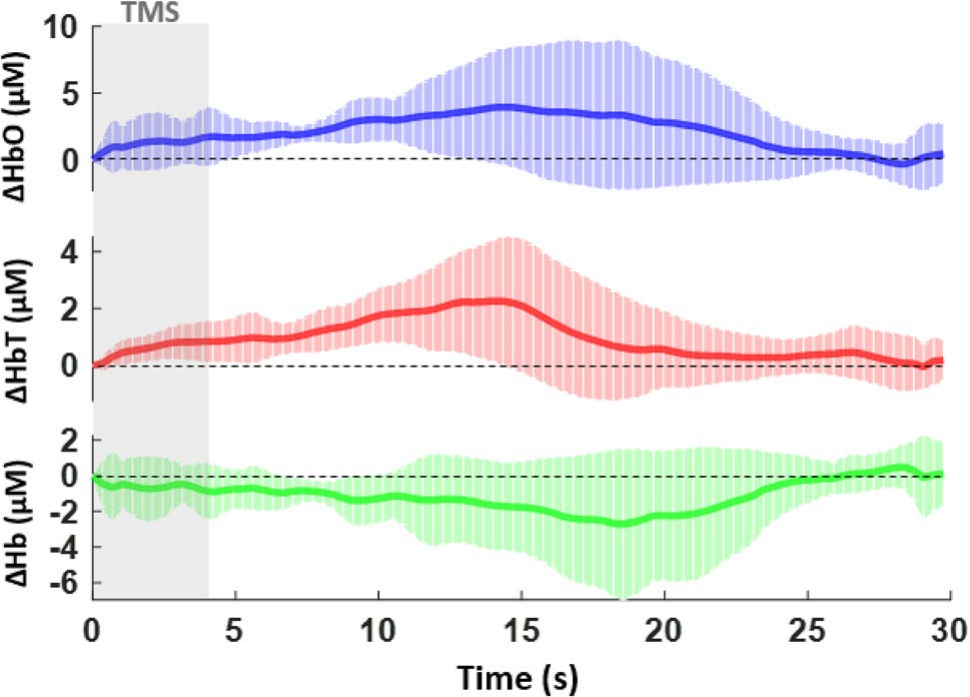
Averaged time course of oxygenated [HbO], total [HbT], and deoxygenated [Hb] hemoglobin signals in the depressed subjects for the contralateral or right dorsolateral prefrontal cortex. Ten hertz stimulation was performed for 4 s followed by 26 s of rest for a total of 12 epochs. The x-axis represents the time from 0 to 30 s during the epoch and the y-axis represents the mean and standard deviation for relative hemoglobin concentration in µM/L.

As hypothesized, there was an observable quantitative difference in the hemodynamic response between healthy and depressed subjects within the right dorsolateral prefrontal cortex. In healthy individuals, [HbO] and [HbT] began to increase sharply beginning at 2.62 s until a peak observed at 10.40 s (95% CI [1.544, 9.609] and [1.734, 6.220] for [HbO] and [HbT], respectively). The average absolute value of hemoglobin change was calculated to be 5.58 µM and 3.98 µM for [HbO] and [HbT], respectively. [Hb] was found to decrease in a reciprocal manner at first until 5.3 s, with a gradual decline thereafter for the rest of the epoch. Its average absolute value was calculated at 3.04 µM (Fig. 2). In the depressed subjects, [HbO] and [HbT] increased more gradually and their values plateaued later in comparison to the healthy subjects at 14.55 s (95% CI [1.869, 4.169] and [1.108, 2.462] for [HbO] and [HbT], respectively) (p = 0.013 for [HbO] and p = 0.011 for [HbT]). The average absolute values of hemoglobin change in this group were noted to be decreased, at 3.98 µM and 2.27 µM for [HbO] and [HbT], respectively. [Hb], in a similar fashion, decreased gradually though reached a nadir later at 18.43 s (Fig. 3). The average value of change was calculated to be 2.71 µM.

When comparing the two groups, the difference in time to peak [HbO] and [HbT] was 4.15 s (p = 0.032 for [HbO] and p = 0.010 for [HbT]), with a later onset in the depressed subjects. The average hemoglobin change in this group was also observed to be decreased by 1.60 µM, 1.71 µM, and 0.33 µM for [HbO], [HbT], and [Hb], respectively.

**Figure 4:**
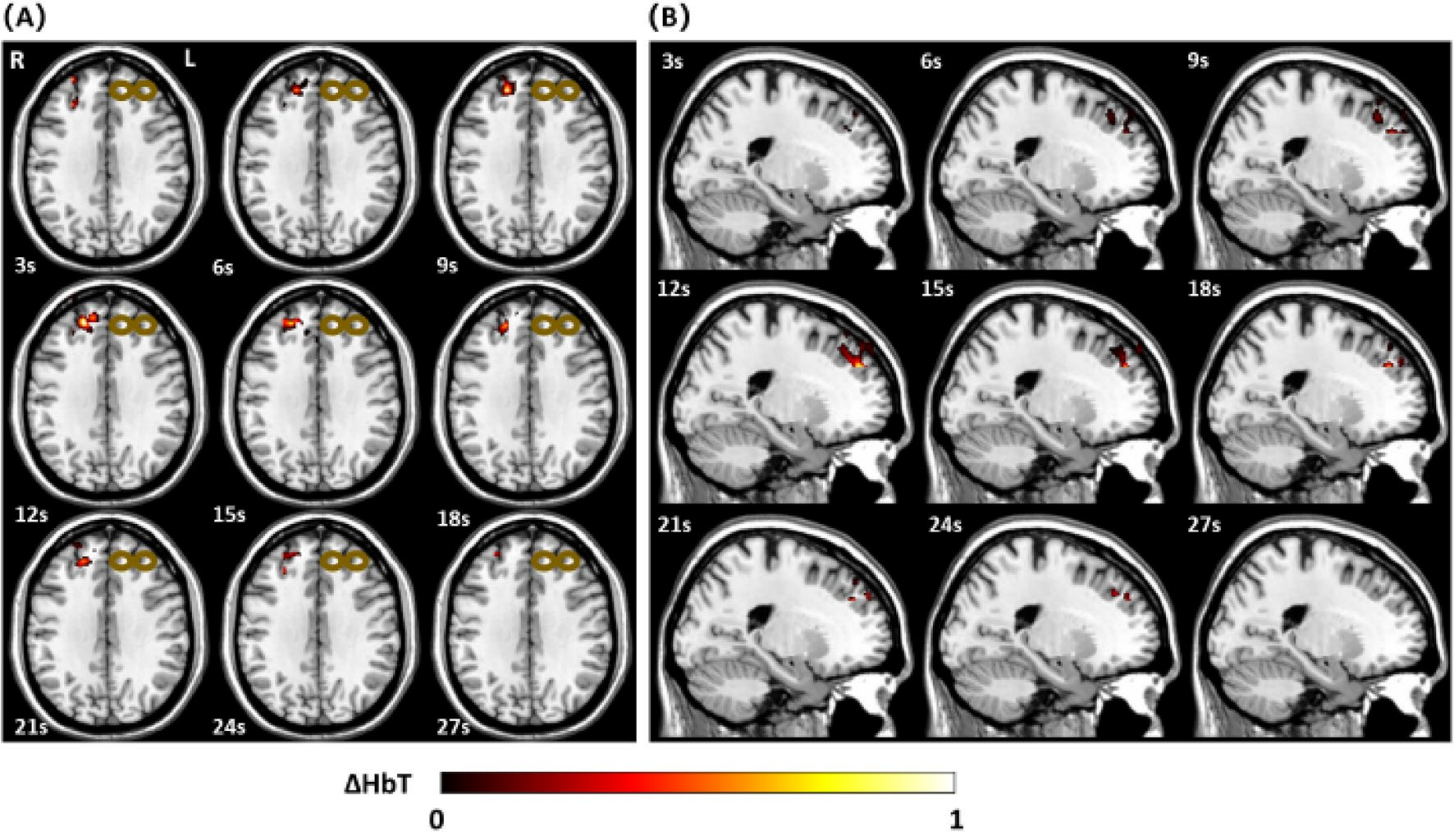
(**A**) Transverse view of the three-dimensional [HbT] images collected by DOT in a healthy subject during a 30 s epoch. (**B**) Sagittal view of the three-dimensional [HbT] images collected by DOT in a healthy subject during a 30 s epoch. Data was only acquired from the right hemisphere of the brain. The bronze colored coil symbol represents stimulation of the left side.

**Figure 5:**
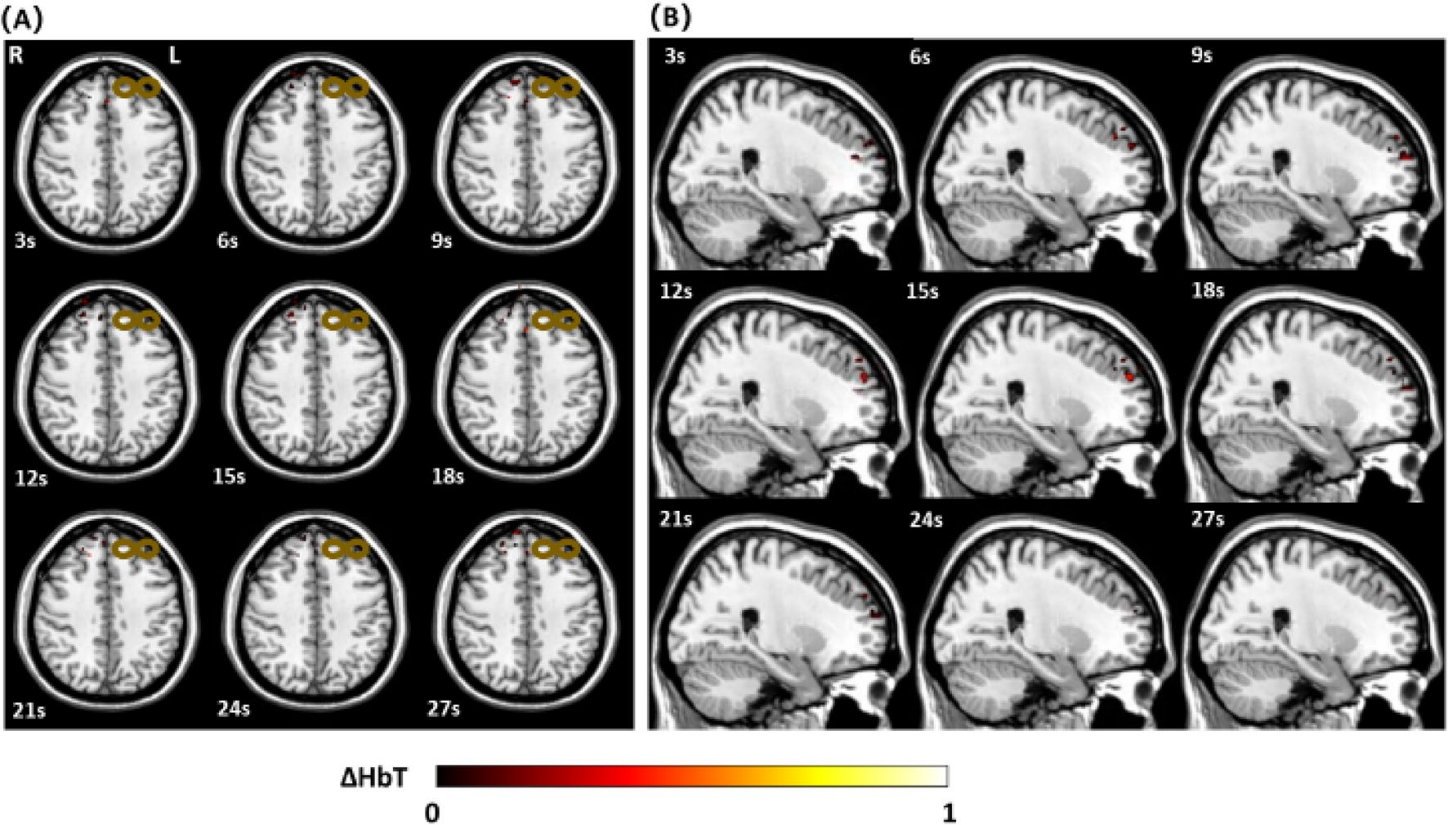
(**A**) Transverse view of the three-dimensional [HbT] images collected by DOT in a depressed subject during a 30 s epoch. (**B**) Sagittal view of the three-dimensional [HbT] images collected by DOT in a depressed subject during a 30 s epoch. Data was only acquired from the right hemisphere of the brain. The bronze colored coil symbol represents stimulation of the left side.

**Figure 6:**
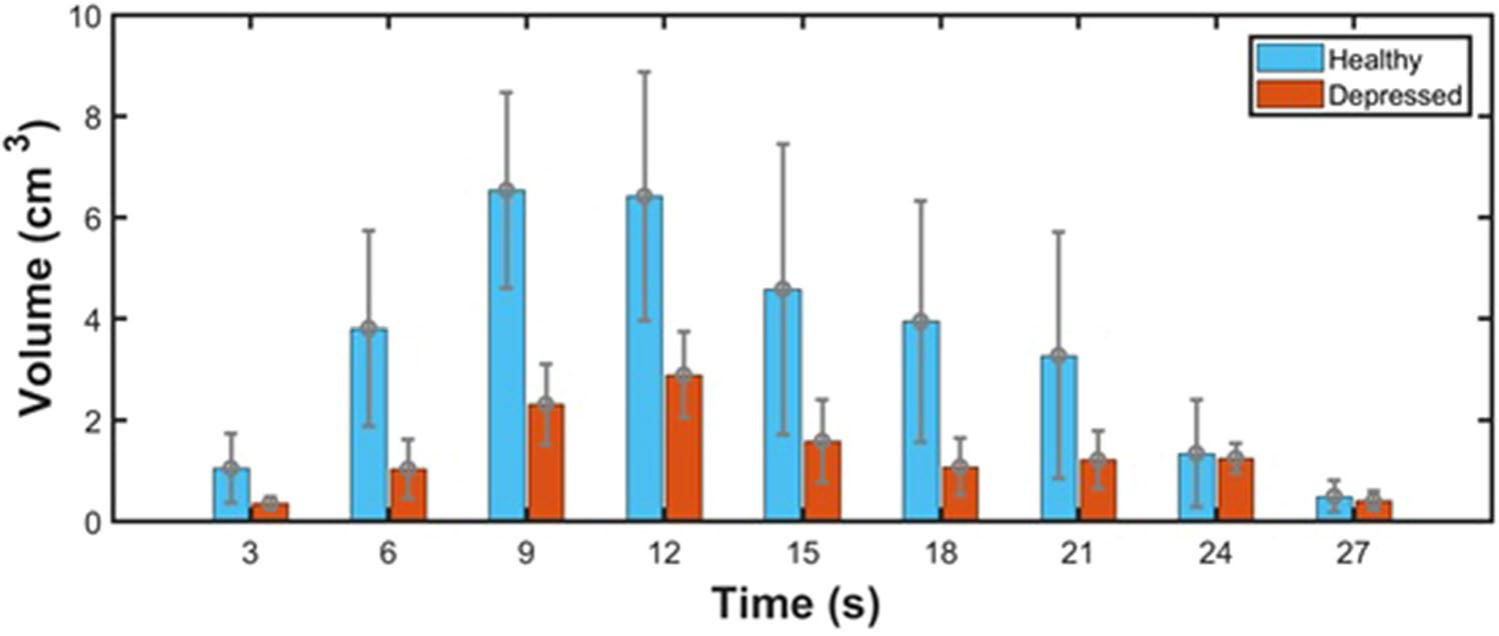
Averaged volume change (in cm^3^) with error bars of [HbO] signal compared between the healthy and depressed groups.

Three-dimensional functional imaging during and after rTMS stimulation was successfully captured by our DOT system. In the provided transverse and sagittal images of the right DLFPC (Figs. 4, 5), clear distinctions in activation patterns between a depressed and healthy subject can be observed. Healthy subjects were observed to have a more robust area of activation prior to a return to baseline. In comparison, depressed individuals had a smaller change in hemodynamic response with a more restricted distribution pattern. By taking advantage of the inherent ability of DOT to assess depth, a volumetric analysis of the change in [HbO] was also calculated. During the 4 s of stimulation, alterations in the volume (in cm^3^) of activation were observed to occur, which continued even after cessation of stimulation. Healthy subjects reached a maximum average volume change of 6.99 cm^3^ whereas depressed subjects peaked at 2.95 cm^3^ (p = 0.016). As can be seen in Fig. 6, we were able to concurrently plot volume changes with three dimensional images in our subjects. An increase in volume stimulated can clearly be seen in both groups that peaks and then gradually trends towards baseline, with a smaller volume of response in the depressed group.

## Discussion

In this study, we have demonstrated for the first time the ability of a novel DOT system to capture cerebral hemodynamic activity during and after rTMS in depressed and healthy individuals. Previous studies with fNIRS and other modalities have only been able to offer incomplete insights due to the lack of adequate temporal and spatial resolution, and more importantly, volumetric three-dimensional images. Many of those experiments focused on lower frequency stimulation in healthy subjects as well. No prior study has directly compared depressed individuals with healthy ones, especially using treatment parameters closer to those that are approved by the FDA in the United States. As such, the implications of our findings potentially create a new frontier in furthering our knowledge of the mechanism of TMS in depression and how we can improve it to more effectively treat depression.

A plethora of past fNIRS studies have observed how different frequencies and motor thresholds of TMS stimulation cause variable inhibitory or excitatory effects within the brain. Lower frequencies (1, 2, 5 Hz) tend to cause decreases in [HbO] in the motor or prefrontal cortices, and previous studies have demonstrated that 1 Hz stimulation on the right DLPFC has a clinical effect on treating depression. 10 Hz stimulation has been shown to increase [HbO], which was associated with continued therapeutic effects of TMS^34–36^. Our results show a clear difference in the time to peak [HbO] and [HbT] and average hemoglobin value change between depressed and healthy subjects. There is a noticeable delayed and decreased hemodynamic change that likely reflects a less robust response to rTMS treatment. The reasoning underlying this finding is possibly linked to what is occurring at the neuronal or cellular level.

The neurobiology and pathophysiology of depression is a much-debated topic without any definitive conclusions. There is suspected to be a complex interplay within multiple realms including neurocircuitry, molecular signaling, genetics and epigenetics, homeostatic adaptations, and immune responses^37^. An exact circuit has not been identified, though several regions have been noted to be malfunctioning including the orbitomedial prefrontal cortex, anterior cingulate cortex, amygdala, hippocampus, cerebellum, and basal ganglia^38^. Within these areas, one such hypothesis suggests that there are redundant and dysfunctional firing of neurons which leads to overall decreased activity, and thus reduced neurovascular coupling and blood flow, which is potentially what is being observed in our depressed subjects^39^.

Regarding laterality of stimulation in relation to our results, previous studies have demonstrated variable results. Some report bilateral and congruent responses, whereas others report interhemispheric differences^18^. The underlying etiology for these discrepancies is unknown. Proposed hypotheses have included disjointed coupling and heterogeneous connectivity errors in the setting of depression through pathoetiologic mechanisms as described above. An additional reason may be due to the varying capabilities of previous imaging modalities and diversity of their sample sizes. Though we were unable to study the ipsilateral DLPFC (due to technical feasibility), our results of the contralateral side were congruent with the majority of previous fNIRS literature^18^. Moreover, previous studies have not been able to reliably capture three-dimensional data during the active stimulation in both depressed and healthy subjects, thus highlighting another potential explanation for any differences observed here compared to other studies.

In terms of our volumetric three-dimensional analyses, we also observed distinct differences in the time to maximum volume change between depressed and healthy subjects. At 12 s (or 8 s after the stimulation window), depressed subjects only reached approximately half the depth of hemodynamic change that healthy individuals experienced. Although DOT is unable to reliably obtain data of the deepest structures within the brain, this finding may further reinforce the rationale underlying theories about what TMS is truly stimulating in depression. The left DLPFC is the most common target for treating depression not only necessarily due to this specific region itself, but from studies demonstrating that it modulates the blood flow response and activity of the anterior cingulate cortex as well^40^. The rates of perfusion and metabolism in this region in particular may predict response to treatments^41^. Thus, the observed reduced hemodynamic response of the DLPFC may suggest that either: (1) even near-FDA approved treatment parameters in our study were not adequate enough to generate appropriate treatment response or stimulation or (2) depressed individuals at baseline have a blunted response and could require individualized treatment parameters to achieve proper activation. However, this would have to be clarified in future studies that directly study the connectivity between the DLPFC and anterior cingulate cortex or other structures actively during stimulation.

The significance of our data then may potentially pave the way towards truly optimizing TMS for depression at even the individual level. Ideal stimulation parameters have remained unknown as there has previously been no reliable mechanism to measure neural connectivity and hemodynamic responses actively during stimulation. Utilizing DOT simultaneously with TMS in testing variable frequencies, intensities, number of pulses, and interval timings offers a new promising approach to allowing such exploration in real time and with three dimensional images. Cortical activation/inhibition patterns and hemodynamic responses would be reliably captured in depressed and healthy individuals. In addition, pairing DOT with newer protocols such as intermittent theta burst stimulation (iTBS) may also lend further credence to such approaches. iTBS uses 50 Hz triplet “bursts” every 200 ms to activate the brain and deliver an entire therapeutic dose equivalent of stimulation in 3 to 10 min versus the standard 18 to 37 min. Recently Cole et. al published the first trial demonstrating a high efficacy when delivering iTBS ten times daily for 5 days, thus dramatically shortening the usual treatment time^42^. To the best of our knowledge, no neuroimaging studies have been conducted which compare iTBS or other techniques with traditional 10 Hz rTMS, which a direct comparison may offer even further insights into discovering optimal parameters. Additionally, using a modality like DOT to study iTBS by itself may help us understand this particular technique more as well.

Another unanswered question of how TMS can be improved involves developing more precise stimulation site targeting. Traditionally, providers will identify the left DLPFC by the “5-cm rule”, which involves finding the area over the motor cortex that produces a finger twitch, then moving 5–6 cm anterior^43,44^. As there are inherent differences in neuroanatomy, this method may lead to enormous treatment response heterogeneity among individuals^45^. It has been recently speculated that structural magnetic resonance imaging-guided placement of the coil may lead to improved outcomes; however, this has not been established^46^. Instead, targeting with fMRI has preliminarily shown improved antidepressant response if stimulated at more anterior and lateral locations within the DLFPC or at a site more negatively correlated to the subgenual cingulate^47,48^. Unfortunately, it is very challenging to reliably use fMRI simultaneously with TMS. Additionally, these machines are quite expensive, not portable, and not readily available in many areas of the world. DOT on the other hand would be able to theoretically assist with targeting in a similar fashion as the area that requires identification is superficial enough to be within standard DOT imaging depths.

Our study was certainly not without limitations. First, this was a pilot study with a heterogeneous, small sample size at a single center, without randomization or a TMS sham group. As such, the generalizability of our results is limited due to the inherent design. Additionally, due to the small sample size, there was a notable imbalance between the study groups in terms of age and gender. As such, age and gender-related cortical atrophy could have affected our results given potential changes in coil to cortex distance theoretically^49^; though the average age of our groups was not within the range at which atrophy rates increase significantly. We also did not conduct any regular standardized symptom scales or assessments throughout that would allow us to comment on TMS treatment efficacy differences within the depressed group. Next, the intensity of our TMS parameters was set at 100% instead of 120% which could certainly affect the neural activation patterns captured by imaging. Our parameters otherwise were within FDA approved limits for the treatment of depression. For initial feasibility purposes, the DOT setup was created to obtain images from the contralateral DLPFC. Directly under the coil and bilateral data acquisition would have been more ideal, as fNIRS can be used in such a way. However, our results were still congruent with previous functional imaging data and future directions include augmentation of the setup to obtain bilateral information in subsequent studies.

In addition, further obstacles regarding the DOT setup itself should be mentioned. Due to the physical limitations of the optical fibers and optodes used in the headgear design, meticulous planning of the array must be considered. Movement artifacts, as with any other techniques, is still a significant barrier that will inevitably be present. Proper construction of the optode headgear with fixation may assist with reducing this phenomenon, along with further advancements in post-processing and filtering methodologies. Physiological signal contamination is another technical limitation that is common to all optical methods especially. Although DOT suffers much less from scalp interference (compared to fNIRS), the presence of hair itself or dark shades impairs signal quality. As such, pinning or separation of hair is an unideal and imprecise method often employed. Further development of analysis and filtering algorithms may assist with bypassing this issue. Additionally, though theoretically the magnetic field of TMS should not interfere with the DOT recording signal, there are no published studies of this (likely due to the improbable nature of it occurring). Lastly, the maximum depth that can be reliably and accurately imaged is greater than fNIRS, though is still limited compared to fMRI. It is estimated to be approximately 5 cm, which reduces the ability to detect deeper brain structures (e.g., basal ganglia, amygdala), but more than capable of capturing cortical and some subcortical structures.

## Conclusion

In summary, this is the first study to our knowledge of using DOT to simultaneously measure functional brain changes induced by rTMS in depressed and healthy subjects. Standard treatment parameters were conducted along with concurrent neuroimaging that demonstrated a delayed and less robust response in depressed individuals. Three-dimensional images with high spatial and temporal resolutions were collected, along with associated volumetric changes. This novel optical neuroimaging device is a safe, noninvasive, portable, and effective modality that may pave the path towards furthering our understanding of neural hemodynamic responses in depression and with further studies may allow for optimization of treatment parameters and thus improved outcomes in depression.

## Supplementary Information

Supplementary Information 1.

Supplementary Information 2.
