# Supplementary material for: Neuroimaging of depression with diffuse optical tomography during repetitive transcranial magnetic stimulation
Source: Sci Rep. 2021 Apr 1;11:7328. doi: 10.1038/s41598-021-86751-9 (PMC8016845; doi:10.1038/s41598-021-86751-9)
Supplement: Supplementary file 1 — Supplementary Information 1. [file 41598_2021_86751_MOESM1_ESM.docx]

**Title:** Neuroimaging of depression with diffuse optical tomography during repetitive transcranial magnetic stimulation

**Authors:** Shixie Jiang^1^, Jingyu Huang^2^, Hao Yang^2^, Ryan Wagoner^1^, F. Andrew Kozel^1,3^, Glenn Currier^1^, Huabei Jiang^2*^

**Affiliations:**

^1^Department of Psychiatry and Behavioral Neurosciences, University of South Florida, Tampa, FL, USA

^2^Department of Medical Engineering, University of South Florida, Tampa, FL, USA

^3^Department of Behavioral Sciences and Social Medicine, Florida State University, Tallahassee, FL, USA

***Corresponding author:**

Huabei Jiang, PhD

University of South Florida

Department of Medical Engineering

4202 E. Fowler Avenue, ENG 030

Tampa, FL 33620, USA

Phone: 813-974-3780

E-mail: [hjiang1@usf.edu](mailto:hjiang1@usf.edu)

**Short title:** Optical imaging of depression during TMS

**Keywords:** diffuse optical tomography, optical imaging, repetitive transcranial magnetic stimulation (rTMS), major depressive disorder, brain imaging


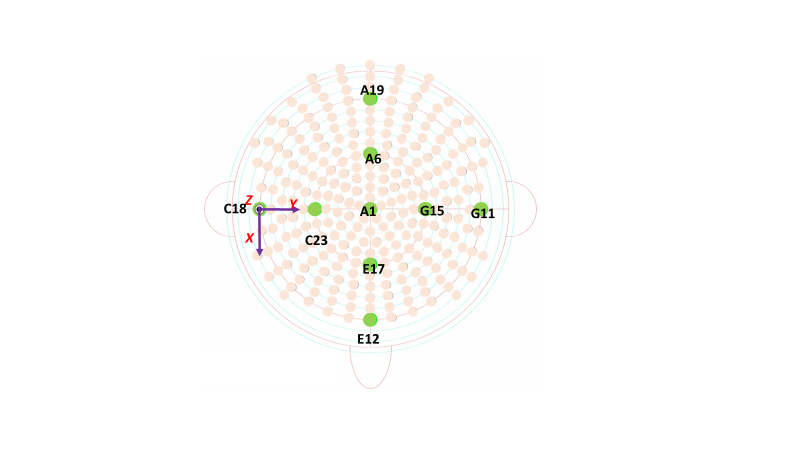


Figure S1. Schematic of the coordinate system used based on a Cartesian three-dimensional configuration combined with a 256 channel EEG system.
