# Supplementary material for: Neuroimaging of depression with diffuse optical tomography during repetitive transcranial magnetic stimulation
Source: Sci Rep. 2021 Apr 1;11:7328. doi: 10.1038/s41598-021-86751-9 (PMC8016845; doi:10.1038/s41598-021-86751-9)
Supplement: Supplementary file 2 — Supplementary Information 2. [file 41598_2021_86751_MOESM2_ESM.docx]

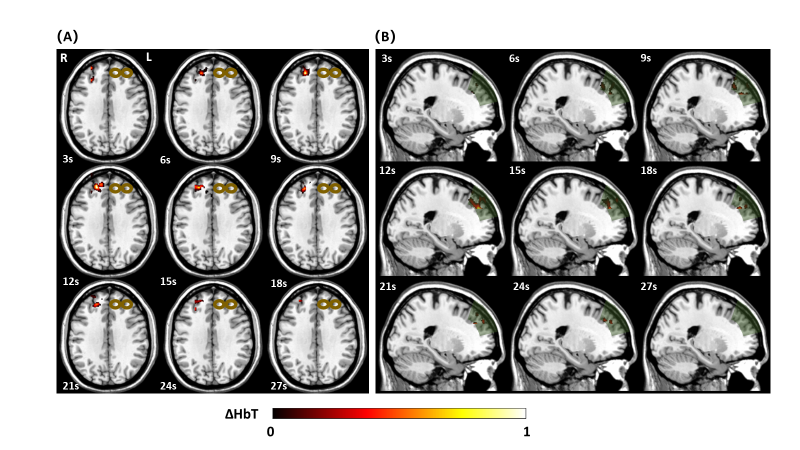


Supplemental Figure 2. A) Transverse view of the three-dimensional [HbT] images collected by DOT in a healthy subject during a 30 s epoch. B) Sagittal view of the three-dimensional [HbT] images collected by DOT in a healthy subject during a 30 s epoch. Data was only acquired from the right hemisphere of the brain. The bronze colored coil symbol represents stimulation of the left side. The dark green shading depicts the region of interest and volumetric capabilities when the coordinates are centered upon the R DLPFC.


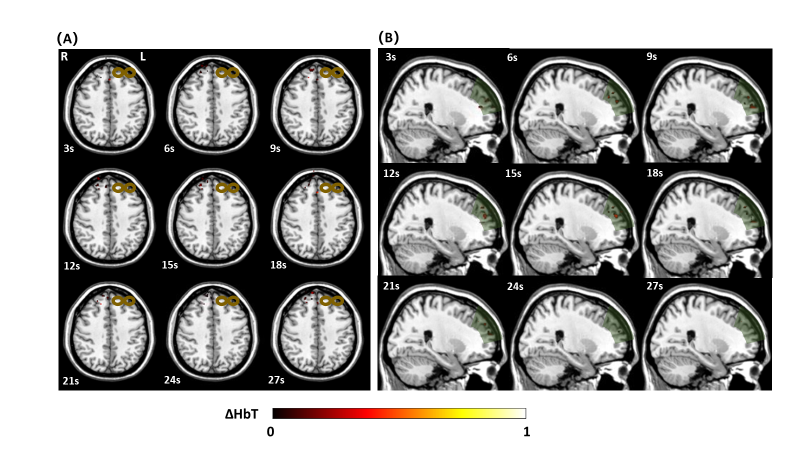


Supplemental Figure 3. A) Transverse view of the three-dimensional [HbT] images collected by DOT in a depressed subject during a 30 s epoch. B) Sagittal view of the three-dimensional [HbT] images collected by DOT in a depressed subject during a 30 s epoch. Data was only acquired from the right hemisphere of the brain. The bronze colored coil symbol represents stimulation of the left side. The dark green shading depicts the region of interest and volumetric capabilities when the coordinates are centered upon the R DLPFC.
